# Tenecteplase: biochemical and clot lysis activity comparisons

**DOI:** 10.3389/fphar.2024.1498116

**Published:** 2024-12-20

**Authors:** Jan Bechmann, Ira Schmid, Simone Brand, Felix Miller, Chengzhi Zhang

**Affiliations:** ^1^ Boehringer Ingelheim Pharma GmbH and Co., KG, Biberach an der Riss, Germany; ^2^ Department of Pharmacy, The First Affiliated Hospital of Chongqing Medical University, Chongqing, China

**Keywords:** tenecteplase, copy, clot lysis, fibrinolysis, glycosylation, thrombosis

## Abstract

**Introduction:**

In the last decades, the recombinant tissue plasminogen activator alteplase has been the standard fibrinolytic treatment of acute myocardial infarction, pulmonary embolism, and acute ischemic stroke. An optimized version of alteplase, tenecteplase, has been developed by exchanging six amino acids to increase half-life, achieve higher fibrin selectivity and increase resistance to plasminogen activator inhibitor-1. Meanwhile, several products containing tenecteplase exist. The aim of this study was to compare the fibrinolytic activity and overall product quality of the 25 mg/vial presentation of tenecteplase originator Metalyse^®^ (Boehringer Ingelheim Pharma GmbH and Co., KG, Ingelheim, Germany) to the 16 mg/vial formulation of the tenecteplase copy Mingfule^®^ (CSPC Recomgen Pharmaceutical, Guangzhou, Co., Ltd.).

**Methods:**

We have systematically analyzed and evaluated the biochemical and fibrinolytic differences between Metalyse^®^ and Mingfule^®^ using a wide range of routine quality testing assays, supplemented by mass spectrometry analysis and surface plasmon resonance assays. Additional host cell protein quantification and clot lysis testing following plasmin incubation over time were performed.

**Results:**

Several key differences in biochemical composition and clot lysis activity were observed between the two tenecteplase variants. Versus Metalyse^®^, Mingfule^®^ exhibited lower clot lysis activity and contained less of the two-chain form of tenecteplase. In addition, there were differences in sialic acid content, galactosylation, and fucosylation patterns, with Mingfule^®^ exhibiting more bi- and less tri- and tetra-antennary glycosylation, leading to a different charge and size heterogeneity profile. Furthermore, Mingfule^®^ displayed highly dissimilar binding to the three clearance receptors (LRP-1, ASGR, and mannose receptor) compared with Metalyse^®^. Purity analysis showed that Mingfule^®^ contained a lower monomer content and, in contrast to Metalyse^®^, substantial amounts of host cell protein.

**Discussion:**

Taken together, these data demonstrate that the tenecteplase copy Mingfule^®^ has several meaningful fibrinolytic and biochemical differences compared with Metalyse^®^. This raises the question of whether data from clinical studies with one of the products can be generalized for all tenecteplase variants.

## 1 Introduction

Thrombotic events and their complications, such as acute myocardial infarction, pulmonary embolism, and acute ischemic stroke (AIS), are leading causes of death globally and are major contributors to the global health burden (Wendelboe and Raskob, 2016). Restoration of blood flow and improvement of perfusion to the affected organ are essential for survival. Therefore, the mainstay of treatment has focused on the breakdown of the clot through thrombolysis with a tissue plasminogen activator (Singh et al., 2023).

The efficient formation of fibrin is critical for hemostasis and wound healing; however, if too much fibrin is produced and/or is deposited in non-bleeding locations, debilitating blood clots may occur. Clot structure is dependent on the properties of individual fibrin fibers, which come together to form a fibrin mesh. Clots differ in their structure in terms of fibrin fiber diameter, density, stiffness, and permeability. Fibrinolysis, specifically the breakdown of fibrin elements of a clot, is the proteolytic degradation of fibrin to facilitate the dissolution and clearance of blood clots from circulation (Weisel and Litvinov, 2014; Mihalko and Brown, 2020; Mutch and Medcalf, 2023). The serine protease tissue plasminogen activator (t-PA), a physiologically occurring glycoprotein, is the main endogenous mediator of fibrinolysis (Mutch and Medcalf, 2023). A recombinant version, rt-PA (alteplase), was the standard fibrinolysis treatment in acute myocardial infarction, pulmonary embolism, and AIS. Tenecteplase has been modified from alteplase at three positions, resulting in an increased half-life, higher fibrin selectivity, and increased resistance to plasminogen activator inhibitor-1 (PAI-1) (Davydov and Cheng, 2001). Therefore, tenecteplase can be administered as a single-dose bolus application during the early stages of an acute myocardial infarction (Llevadot et al., 2001; Melandri et al., 2009). Tenecteplase is licensed for the treatment of ST-elevation myocardial infarction (STEMI), a life-threatening emergency resulting from complete occlusion of a coronary artery by a clot, and for the fibrinolytic treatment of AIS (Boehringer Ingelheim International GmbH, 2024; CSPC Pharmaceutical Group Limited, 2024). Several studies have reported on higher reperfusion rates, similar safety and efficacy, and improved treatment times for tenecteplase in comparison with alteplase (Potla and Ganti, 2022; Singh et al., 2023).

Fibrinolysis and its regulatory mechanisms involve a complex system of biochemical reactions (Weisel and Litvinov, 2014). The activity of tenecteplase depends on fibrin binding and, for efficient conversion of plasminogen to plasmin and subsequent plasmin-driven clot lysis, tenecteplase needs to enter a clot containing fibrin and plasminogen. After activation of plasminogen to plasmin by tenecteplase, plasmin further increases the fibrinolytic activity of tenecteplase by cleaving the single-chain into the two-chain form of the molecule (Tanswell et al., 2002).

It is important to note that several products containing tenecteplase exist. Whilst generic versions of new chemical entities contain the same chemical substance as the original drug (and therefore elicit the same quality and performance characteristics), biologic drugs, also known as biopharmaceuticals, are inherently more complex molecules. This is due to several factors, including structural intricacy and variability, formulation, sensitivity, and immunogenicity. Moreover, the manufacturing process of biologic drugs can significantly influence their properties. For development and production, it is crucial to consider general quality attributes that can significantly influence the performance and safety of biopharmaceuticals. These attributes encompass a wide range of factors, including post-translational modifications, molecule-related impurities, process-related impurities (PRIs), and other product-specific attributes. In the context of PRIs, the presence and quantity of host cell proteins (HCPs) in a biopharmaceutical product can have significant implications for its safety, efficacy, and overall quality. High levels of HCPs can potentially lead to adverse immune responses in patients, impacting the drug’s safety profile. Additionally, certain HCPs may interact with the drug substance, potentially affecting its stability, potency, and overall performance (Wang et al., 2009; Halimi et al., 2020). Therefore, the production of biologic drugs requires meticulous attention to ensure consistency, efficacy, and safety (Blandizzi et al., 2017; Halimi et al., 2020).

Copy versions of tenecteplase include Mingfule^®^ in China, which was approved by China’s National Medical Products Administration in 2015 for use in patients with STEMI, and is also approved for AIS (CSPC Pharmaceutical Group Limited, 2015; CSPC Pharmaceutical Group Limited, 2024).

This study aimed to compare the fibrinolytic activity and overall product quality of the original tenecteplase, Metalyse^®^ 25 mg/vial presentation (Boehringer Ingelheim Pharma GmbH and Co. KG, Ingelheim, Germany), to the 16 mg/vial formulation of the tenecteplase copy Mingfule^®^ (CSPC Recomgen Pharmaceutical, Guangzhou, Co., Ltd.).

## 2 Materials and methods

All release testing was performed using International Conference on Harmonisation of Technical Requirements for Registration of Pharmaceuticals for Human Use validated methods according to standard operating procedures, which are applied to the routine quality control testing of tenecteplase for market release of batches produced by Boehringer Ingelheim. An overview of intermediate precision of methods is given in Table 1. Testing was supplemented by state-of-the-art mass spectrometry (MS) analysis and surface plasmon resonance (SPR) measurements (an optical biosensing technique to study molecular interactions in real time). Furthermore, HCP quantification and clot lysis testing following plasmin incubation over time were performed.

**TABLE 1:** Intermediate precision of Metalyse^®^ release methods.

Test method	Intermediate precision coefficient of variation (%)
Clot lysis release assay	≤3
Single-chain determination by HP-SEC	≤1
Monomer content by HP-SEC	≤1
CGE reduced	FR1: 4.0 MP: ≤1
icIEF	Region 1: ≤1 Region 2: ≤1 Region 3: ≤2.2
Host cell proteins by CHOP-ELISA	≤10
Type I/II determination by RP-HPLC	≤1

CGE, capillary gel electrophoresis; CHOP-ELISA, Chinese hamster ovary cell protein-enzyme-linked immunosorbent assay; FR1, fragment region 1; HP-SEC, high-performance size exclusion chromatography; icIEF, imaged capillary isoelectric focusing; RP-HPLC, reversed-phase high-performance liquid chromatography.

### 2.1 Drugs to be investigated

Three batches each of Metalyse^®^ 25 mg/vial and Mingfule^®^ 16 mg/vial (exclusively available in China) were analyzed. Both drugs were stored at the recommended storage conditions and were well within the expiration date (7–8 months for Metalyse^®^ batches, and 8–13 months for Mingfule^®^ batches). Following the respective instructions for use, both drugs were reconstituted with water for injection, yielding solutions with concentrations of 5.1 mg/mL for Metalyse^®^ and 5.4 mg/mL for Mingfule^®^. Samples were reconstituted in the same time frame and stored at −20°C until analytical testing was performed. The analytical testing was conducted based on the actual protein concentration in each solution – i.e., the same amount of protein was used allowing a direct comparison of results between Metalyse^®^ and Mingfule^®^.

### 2.2 Functional assays

#### 2.2.1 Preparation of reagents

Thrombin (Calbiochem, cat. #605195-1000U) was reconstituted in 1 mL G-water (molecular grade water) and on the day of analysis diluted to 33 U/mL in assay buffer (0.0114 mol/L NaH_2_PO_4_ × 2H_2_O, 0.0486 mol/L Na_2_HPO_4_ x 2H_2_O, 0.02% [m/V] NaN_3_, 0.01% [V/V] 10% Tween 80). Fibrinogen (Calbiochem, cat. #341578) was reconstituted in assay buffer to 2 mg/mL, incubated at 37°C with infrequent gentle mixing to facilitate dissolution, and filtered through a pleated filter. Plasminogen (Calbiochem, cat. #528185-1MG) was reconstituted in G-water to a final concentration of 1.5–1.9 mg/mL. Each clot lysis assay included freshly prepared reference standard and a positive control sample of the current material for release testing.

#### 2.2.2 Clot lysis analysis

*In vitro* clot lysis activity of Mingfule^®^ and Metalyse^®^ samples was determined using an automated analysis system as described before (Kliche et al., 2014).

Plasmin-induced potency activation of Metalyse^®^ and Mingfule^®^ was carried out using plasmin (Calbiochem cat. #527621-10U), which was freshly diluted at a ratio of 1:25 in G-water prior to use. For the plasmin-induced conversion of single-chain to two-chain form, followed by clot lysis analysis, the Metalyse^®^ or Mingfule^®^ samples were washed with the Metalyse^®^ formulation buffer using 30 kDa Amicon centrifuge filters. A 500 µL sample volume (5 mg/mL) was mixed with 2 µL of human plasmin (concentration: 2.32 mg/mL; specific activity 22.6 U/mgP) and incubated at 37°C and 300 rpm on a shaking heater for a maximum of 6 h. At multiple time points, 20 µL of each sample was mixed with 780 µL dithiothreitol (DTT) to stop the enzymatic conversion. The Metalyse^®^ and Mingfule^®^ samples were denatured by incubating at 80°C for 8 min. For each measurement time point, 20 µL of the reaction mixture was diluted in assay buffer to a concentration of 32 μg/mL and subsequently analyzed using the ACL TOP^®^ system according to the published standard procedure. The effect of plasmin on the clot lysis activity was assessed. No clot lysis activity was observed in the presence of plasmin but absence of Metalyse^®^/Mingfule^®^.

### 2.3 SPR fibrin binding assay

#### 2.3.1 Preparation of reagents

Fibrinogen (Merck/Calbiochem; cat #341578) from human plasma was reconstituted in sodium phosphate buffer (0.06 M, pH 7.4) to a final protein concentration of 2 mg/mL. The solution was incubated at 37°C for approximately 30 min until the solution was completely clear. The solution was filtered through a pleated filter to remove possible precipitates and stored at 2°C–8°C or on ice until use. Thrombin from human plasma (Merck/Calbiochem, cat #605195) was dissolved in 1 mL purified water and diluted to 60 U/mL in HBS-EP+ (10 mM HEPES, 150 mM NaCl, 3.4 mM EDTA, 0.05% Tween 20; pH 7.4), termed Component A. Coagulation factor XIII from human plasma (Merck/Calbiochem, cat #233501) was diluted in Ca-HBS (HBS-EP+ mixed 1:1 with 4 M CaCl_2_ [Carl Roth, cat #CN93.2]) to a final protein concentration of 50 μg/mL, termed Component B. Solutions of fibrinogen, thrombin, and factor XIII were freshly prepared and used the same day.

#### 2.3.2 Preparation of a ready-to-use SPR-Biosensor chip and binding analysis

All SPR-based measurements were determined using a Biacore T200 analytical system (Cytiva) with the analysis temperature set to 25°C. SPR system and dilution buffer HBS-EP+ and SPR-Biosensor chips (CM5, cat #BR-1005-30) were purchased from Cytiva.

Briefly, fibrinogen was immobilized on the sensor chip surface using (1-Ethyl-3-[3-dimethylaminopropyl]carbodiimide, hydrochloride)/N-hydroxysuccinimide (EDC/NHS) amine coupling (Cytiva, cat #BR100050). For this, fibrinogen was diluted in 10 mM sodium acetate, pH 5.5, to a final protein concentration of 100 μg/mL. An immobilization level of 12,000 resonance units (RU) was targeted by applying the immobilization wizard (aim for immobilized level) of the Biacore Control software. Unreacted, activated carboxyl groups were subsequently deactivated using ethanolamine. The immobilized fibrinogen was then cross-linked by 20 injections of a 1:1 mixture of Components A (thrombin) and B (coagulation factor XIII). Injections of this mixture were performed at a flow rate of 5 μL/min and with a contact time of 360 s. After the last injection, the matrix was conditioned with 1x HBS-EP+ at a flow rate of 50 μL/min for 3 h. A blank immobilization using EDC/NHS and subsequent deactivation with ethanolamine was performed to prepare the reference flow cell.

Before measuring a sample, the matrix was conditioned using five injections of 10 mM HCl (regeneration solution) at a flow rate of 30 μL/min, a contact time of 24 s each, and a stabilization time of 60 s after each injection. During the assay run, each injection was performed with a flow rate of 5 μL/min, with a contact time and dissociation time of 120 s each. Regeneration of the chip involved three injections of 10 mM HCl at a flow rate of 30 μL/min and a contact time of 24 s each, as well as a final stabilization time of 120 s.

Binding curves were double-referenced by first subtracting nonspecific binding from a reference flow cell and then subtracting a blank cycle where buffer was injected instead of protein sample. These were then analyzed using the sensorgram comparison module of the Biacore T200 evaluation software (ver. 3.2) as described earlier (Karlsson et al., 2016; Gassner et al., 2018). Three different Metalyse^®^ lots, each measured in duplicate and in three concentrations (2, 3, and 4 μg/mL), were used to define a comparison corridor (average ± 3 standard deviation [SD] approach with normalized sensorgrams). Mingfule^®^ lots, measured under identical conditions, were then compared (association and dissociation) to this corridor, yielding a similarity score (Karlsson et al., 2016).

### 2.4 Purity analysis

#### 2.4.1 Chinese hamster ovary cell protein (CHOP)-enzyme-linked immunosorbent assay (ELISA)

The quantification of the HCP CHOP was performed by two ELISA methods using polyclonal CHOP antibodies. The Metalyse^®^-specific assay (goat anti-CHOP, prepared in-house) and a commercially available assay (CHO HCP ELISA Kit, 3G [F550-1], Cygnus) were utilized. All assays included spike and assay controls, and a polyclonal, horseradish peroxidase-coupled antibody was used with fluorescence detection of absorption at 450 nm and reference signal at 630 nm. Fluorescence signals were converted to units, with 1 U corresponding to 1 ng of CHOP standard.

#### 2.4.2 MS-based HCP analysis

The MS-based HCP analysis was performed by subjecting samples to shotgun tandem mass spectra (MS/MS) analysis for unbiased protein identification, with the proteins being diluted, digested, and separated based on hydrophobicity before being subjected to the mass spectrometer. Interfering buffer components were removed through precipitation with trichloroacetic acid, washing with acetone, and resuspension in 8 M urea buffer. The samples were then reduced, carbamidomethylated, and proteolytically cleaved using trypsin. HPLC-ESI-MS and -MS/MS mass spectra were obtained using a UHPLC system coupled to an Orbitrap mass spectrometer, with peptides separated using a specific gradient and recorded in positive ion mode. The trap column was a reversed-phase (RP) chromatography column with 0.5 cm and an inner diameter of 300 μm. The separator column used was an EASY Spray PepMap Neo column (75 μm × 500 mm, 2 μm, 100 Å pore size, Thermo Fisher Scientific). Eluents were A: water/0.1% formic acid; B: acetonitrile/0.1% formic acid. The peptides were separated using a gradient that started from 2% B to 3% B in 2 min, then from 3% B to 23% B in 45 min, and finally from 23% B to 45% B in 15 min at 45°C with a flow rate of 0.25 μL/min. The eluted peptides from the analytical column were subjected to positive ionization at 1.5 kV using the EASY-Spray™ Source (ThermoFisher Scientific, Waltham, MA, United States) of an Exploris 480 mass spectrometer. The mass spectrometer was operated in data dependent acquisition (DDA) mode with survey scans acquired from m/z 375 to 1,200 in the Orbitrap analyzer at a resolution of 120,000, followed by frag mentation of the 20 most abundant ions. MS/MS were obtained using higher-energy collisional dissociation (HCD) at 30%. The isolation window was set at 2 m/z, the Orbitrap resolution at 15,000, the target value at 5E4, and the maximum injection time set at auto. Selected precursor ions for fragmentation (including charge state 2–5) were excluded for 45 s, and the repeat count was set at 1. Protein identification and quantification were achieved using Proteome Discoverer 3.0 and Progenesis QI for Proteomics, respectively, with a false discovery rate below 0.05 on peptide spectrum match and 0.01 on protein level, and only proteins identified in all three technical replicates were quantified.

#### 2.4.3 Monomer content quantification

The monomer and aggregate content were determined using high-performance size exclusion chromatography (HP-SEC). HP-SEC separates molecules according to their molecular size on a SEC column with a porous column matrix (Tosoh Bioscience). Larger molecules have a shorter residence time in the pores and elute first from the column, whereas smaller molecules are seen in the chromatograms as peaks with a greater retention time as they elute later. With this method, the monomers can be separated from high-molecular-weight species (HMW = aggregates). For analysis, an isopropanol/L-arginine/ammonium sulfate buffer at pH 7.3 was used as mobile phase with an isocratic flow of 0.5 mL/min. Analysis was monitored at 280 nm. The relative monomer and aggregate content were calculated as a percentage of the total peak area values.

#### 2.4.4 Single-chain determination by HP-SEC

The percentage of the single-chain variant in the samples was determined by HP-SEC under reducing conditions. The tenecteplase molecule exists as single- and two-chain variants due to an internal clipping site between amino acids arginine 275 and isoleucine 276. The two resulting variants of the molecule are linked by disulfide bridges that are reduced by the addition of DTT. Due to the differences in the molecular weight, single- and two-chain variants can be separated by HP-SEC using a SEC column with a porous column matrix (Tosoh Bioscience). For analysis a sodium dihydrogen phosphate monohydrate/sodium dodecyl sulfate buffer at pH 6.8 was used as mobile phase with an isocratic flow of 0.6 mL/min. Samples were reduced by mixing with DTT and incubated at +80°C for 8 min. Analysis was monitored at 214 nm. The relative content of the single-chain variant was calculated as a percentage of the total peak area values.

### 2.5 Glycosylation analysis

#### 2.5.1 Glycosylation analysis using MS

MS-based analysis was used to determine the relative abundance of N- and O-glycans. Samples were denatured in guanidinium hydrochloride, reduced using dithiothreitol, alkylated iodoacetic acid, and finally digested using trypsin at pH 7.4. The resulting peptides were subjected to C18 (XSelect Peptide CSH C18 Column, Waters) reversed-phase ultra-performance liquid chromatography (RP-UPLC, Acquity, Waters) coupled to high-resolution MS (Q-Exactive, Thermo Scientific) including high-energy collision-induced fragmentation. Fragmentation data and accurate precursor mass were used to identify glycopeptides. For data processing (Byos V4.2, Protein Metrics Inc.), selected ion chromatograms (SICs) of all relevant N- and O-glycopeptides were generated. Relative site-specific quantitation was conducted for each respective glycan by dividing the relative SIC area of the glycan by the sum of SIC areas of all glycans at the specific glycosylation site.

#### 2.5.2 N-glycosylation occupancy: Type I/II determination

Two major N-glycosylation variants of tenecteplase are present: type I and type II. Type I is glycosylated at asparagine (Asn) positions Asn103, Asn184, and Asn448, whereas type II is glycosylated at positions Asn103 and Asn448. For the determination of type I and type II content, samples were cleaved enzymatically by plasminogen, reduced with DTT, and separated by RP-HPLC using a C8 column (Agilent). Eluent A (0.1% trifluoroacetic acid in water) and eluent B (0.1% trifluoroacetic acid in acetonitrile) were used as mobile phases. For elution, a gradient with increasing acetonitrile content (20%–37% eluent B) in the mobile phase at a flow rate of 1 mL/min was applied. Quantitation of type I and type II variants was performed by ultraviolet detection at 214 nm with area percentage evaluation.

### 2.6 Impact on clearance receptors

#### 2.6.1 SPR clearance receptor binding assay (LRP-1, ASGR, MR)

Recombinant low-density lipoprotein receptor-related protein 1 (LRP-1) Cluster IV (cat #5395-L4, R&D), asialoglycoprotein receptor 1 (ASGR1, P01, cat #H00000432-P01, Abnova), and mannose receptor C, type 1 (MRC1, cat #2534-MR, R&D) were reconstituted according to the manufacturers’ instructions. CaCl_2_ was purchased from Merck (cat #1.02392.1000). All SPR measurements were performed using a Biacore T200 (Cytiva) with the analysis temperature set to 25°C. SPR running and dilution buffer HBS-EP+ and SPR-Biosensor chips (CM5, cat #BR-1005-30) were purchased from Cytiva. The standard running buffer HBS-EP+ was supplemented with 8 mM CaCl_2_ if not otherwise specified. Prior to immobilization, all receptors were diluted in 10 mM sodium acetate buffer (pH 4.5) to a final protein concentration of 35 nM. LRP-1 (approx. 1300 RU), ASGR1 (approx. 1500 RU), and MRC1 (approx. 13,000 RU) were then immobilized on parallel flow cells using the EDC/NHS amine coupling kit (Cytiva, cat #BR100050). Unreacted, activated carboxyl groups were subsequently deactivated using ethanolamine. For preparation of the reference flow cell, a blank immobilization using EDC/NHS and subsequent deactivation with ethanolamine were performed.

For interaction analysis, Metalyse^®^ (88, 53, 32 μg/mL) and Mingfule^®^ (53, 32, 19 μg/mL) were injected over all flow cells (flow cells 1–4) at a flow rate of 30 μL/min for 240 s, following a dissociation time of 150 s. The surface was then regenerated using a 120 s injection of HBS-EP+ at 30 μL/min (without CaCl_2_). Before each assay run, the matrix was conditioned using 15 injections of reference standard material at 88 μg/mL. Binding curves were analyzed as described in section 2.2.

### 2.7 Measure of heterogeneity

#### 2.7.1 Analysis of post-translational modifications

Post-translational modifications, including methionine and tryptophan oxidation, asparagine and glutamine deamidation, or lysine glycation, were assessed using MS-based analysis. Samples were denatured in urea, reduced using tris(2-carboxyethyl) phosphinehydrochlorid, and alkylated using iodoacetic acid. Digestion using trypsin was carried out at pH 6.0 for succinimide and isoaspartate analysis, and at pH 7.4 for analysis of all other post-translational modifications. Samples were additionally de-N-glycosylated using glycosidase F. The resulting peptides were subjected to C18 (XSelect Peptide CSH C18 Column, Waters) RP-UPLC (Acquity, Waters) coupled to high-resolution MS including high-energy collision-induced fragmentation (Q-Exactive, Thermo Scientific). Fragmentation data and accurate precursor mass were used to identify unmodified and modified peptides. For data processing, SICs of all relevant modified and unmodified peptides were generated (Byos V4.5-53, Protein Metrics Inc.). Relative site- or peptide-specific quantitation was conducted for each modification by dividing the relative SIC area of the respective modified peptide by the sum of SIC areas of the respective modified and unmodified peptides.

MS-based analysis was used to determine the relative abundance of thiols in the samples. The samples were denatured in guanidine hydrochloride and labeled with N-ethylmaleimide (NEM), after which they were reduced using dithiothreitol, alkylated using iodoacetic acid (IAA), and finally digested using trypsin. The resulting peptides were subjected to C18 (XSelect Peptide CSH C18 Column, Waters) RP-UPLC coupled to high-resolution MS including high-energy collision-induced fragmentation (Exploris 240, Thermo Scientific). Fragmentation data and accurate precursor mass were used to identify NEM- and IAA-labeled peptides. For data processing, SICs of all relevant NEM- and IAA-labeled peptides were generated (Byos V4.5-53, Protein Metrics Inc.). Relative site-specific quantitation of free thiols was conducted for each respective peptide by dividing the relative SIC area of the NEM-labeled peptide by the sum of SIC areas of NEM- and IAA-labeled peptides for the specific, Cys-containing peptide.

#### 2.7.2 Imaged capillary isoelectric focusing (icIEF)

Charge heterogeneity was characterized using icIEF. The process involved mixing samples (Metalyse^®^/Mingfule^®^) with carrier ampholytes, additives, and isoelectric point (pI) markers, then separating and focusing the species by a Maurice C System with a fluorocarbon-coated capillary (Bio-Techne GmbH, protein simple) upon voltage application. Reaction buffer with urea, methylcellulose, pharmalyte, phosphoric acid and pI markers was used. Samples were pre-diluted with urea and afterwards mixed with reaction buffer. Analysis was performed at fluorescence exposure time of 10 s. The icIEF system used whole-column imaging with parallel detection of absorbance and native fluorescence to quantitatively monitor the icIEF pattern of proteins, ultimately determining the charge heterogeneity of the samples, which was calculated as a percentage of three region areas.

Charge heterogeneity was further characterized using icIEF after desialylation. Sialic acids were removed using the enzyme sialidase. Samples were mixed with 200 U α2-3,6,8 Neuraminidase (New England Biolabs) and incubated at 37°C before mixing with carrier ampholytes, additives, and pI markers. Then the procedure was continued as described as above.

#### 2.7.3 Capillary gel electrophoresis under reducing (CGEr) conditions

This method separates reduced Metalyse^®^/Mingfule^®^ species such as low-molecular-weight (LMW) and HMW species, as well as Metalyse^®^/Mingfule^®^ main peak based on their respective molecular sizes. The LMW species are located in fragment region 1 (FR1). Samples were denatured with sodium dodecyl sulfate (SDS), reduced with DTT and labeled with a fluorescent dye by incubation. After electrokinetic injection, sample species were separated by size in a bare fused-silica capillary (SCIEX) filled with a SDS polymer that acts as a sieving matrix. Electrophoretic separation was performed by applying an electric field causing the negatively charged smaller protein–SDS complexes to migrate faster through the gel than larger protein complexes. The different sample species were detected by laser-induced fluorescence with the use of a solid-state laser (excitation wavelength: 488 nm; detection wavelength: 600 nm). The relative proportion of sample purity and product-related impurities was determined.

### 2.8 Statistical analysis

All statistics are descriptive in nature. Our study is based on highly standardized and well-validated quality control release assays. All release assays, MS-based characterization, and Biacore LRP-1, ASGR, and MR binding were performed side by side as technical tetraplicates per lot. Biacore fibrin binding was performed side by side as technical duplicates per lot. HCP quantification by the Cygnus kits was performed side by side as a single measurement per lot.

For plasmin-induced conversion and potency activation experiments, three lots of Metalyse^®^ and Mingfule^®^ were analyzed side by side.

## 3 Results

### 3.1 Lysis activity (potency) analysis

#### 3.1.1 Clot lysis release

**FIGURE 1:**
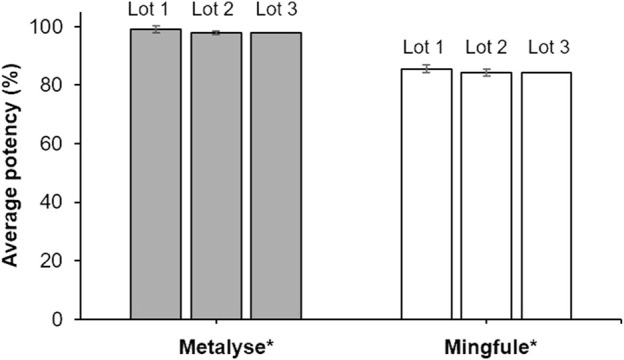
Clot lysis activity (potency) analysis as quantified by clot lysis release assay. *Each lot represents mean ± standard deviation for four replicates.

All three lots of Mingfule^®^ displayed lower clot lysis potency compared with Metalyse^®^, which was used as the reference standard. On average, Mingfule^®^ exhibited 13.5% lower clot lysis potency (84.8%) compared with Metalyse^®^ (98.3%) (Figure 1).

#### 3.1.2 Fibrin binding activity

The individual similarity scores calculated for the three Mingfule^®^ lots were in the range of 37%–53% (Table 2). These results demonstrate that Mingfule^®^ is dissimilar to Metalyse^®^ in fibrin binding, which may partially contribute to the difference in fibrinolytic activity compared with Metalyse^®^.

**TABLE 2:** Similarity of Mingfule^®^ lots to Metalyse^®^ in binding to fibrin and the clearance receptors low-density lipoprotein receptor-related protein 1 (LRP-1), asialoglycoprotein receptor (ASGR), and mannose receptor (MR), measured by surface plasmon resonance.

	Similarity score (%)			
Mingfule^®^ lot	Fibrin	LRP-1	ASGR	MR
Lot 1	53 ± 5	17 ± 2	5 ± 1	8 ± 1
Lot 2	37 ± 2	25 ± 1	6 ± 1	9 ± 0
Lot 3	46 ± 13	30 ± 2	9 ± 2	10 ± 1

### 3.2 Purity analysis

#### 3.2.1 HCP concentration determination

**FIGURE 2:**
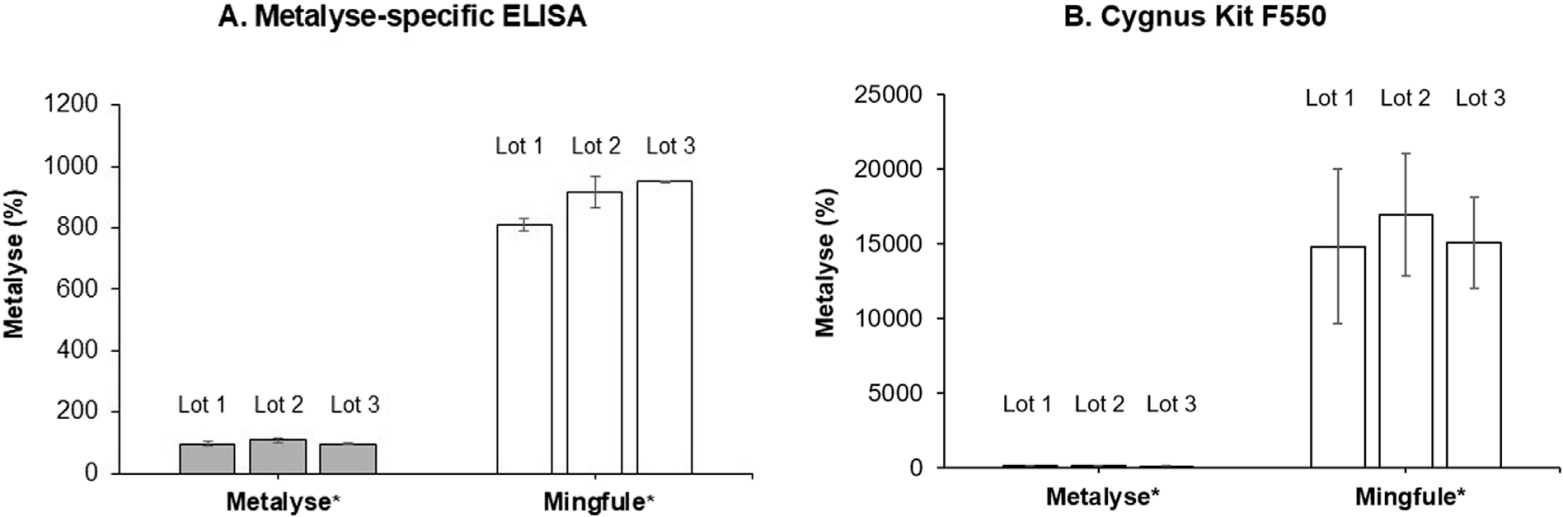
HCP purity analysis measured using two quantification assays. **(A)** Metalyse^®^-specific enzyme-linked immunosorbent assay (ELISA); **(B)** Cygnus CHO HCP 3rd Generation ELISA kit (Cygnus Technologies). Values are given relative to the average of the three analyzed Metalyse lots. *Each lot represents mean ± standard deviation for four replicates

The two HCP quantification assays demonstrated significantly higher HCP content for Mingfule^®^, indicating a lower purity compared with Metalyse^®^ (Figures 2A, B). The HCP content of Mingfule^®^ was up to 185-fold higher than that of Metalyse^®^ depending on the applied assay. In addition, initial MS-based identification of individual HCPs detected a higher number of HCPs and a higher relative abundance of HCPs for Mingfule^®^ (Table 3). In this context, clusterin (data not shown), a protein with immunogenic potential, was detected in all Mingfule^®^ lots, which could not be detected in the Metalyse^®^ lots.

**TABLE 3:** Comparative host cell protein analysis by mass spectrometry.

	Host cell protein content	
	Metalyse^®^	Mingfule^®^
Detected number of HCPs in at least 2/3 lots	43	59
Detected HCPs in at least 2/3 lots specific to the product	3	19
Relative abundance >2-fold higher (HCPs considered that are detected in at least 2/3 lots of both products)	1	8

HCP, host cell protein.

#### 3.2.2 Monomer content quantification by HP-SEC

**FIGURE 3:**
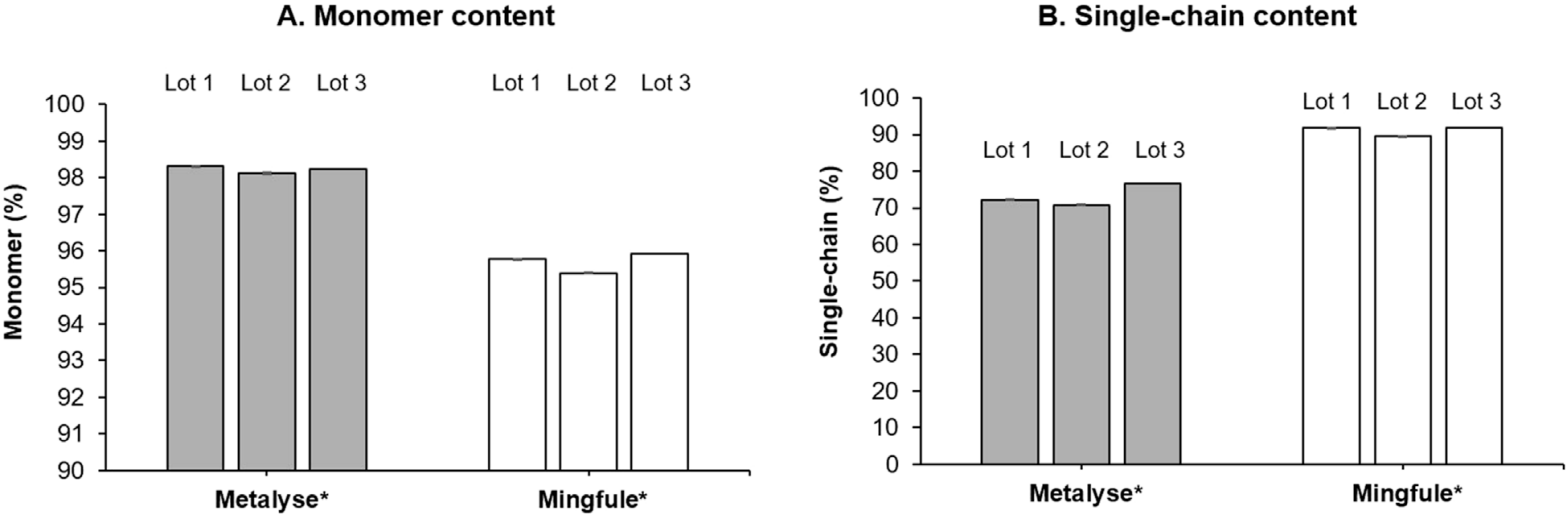
Purity analysis. **(A)** Monomer content; **(B)** single-chain content. *Each lot represents mean ± standard deviation for four replicates

Mingfule^®^ contained a lower monomer content (95.7%, SD 0.3) compared with Metalyse^®^ (98.2%, SD 0.1) (Figure 3A; Supplementary Figure S1). Mingfule^®^ also contained a higher content of HMW species (3.6%, SD 0.2) compared with Metalyse^®^ (1.0%, SD 0.1). These results further suggest that Metalyse^®^ has a higher purity than Mingfule^®^.

#### 3.2.3 Single-chain determination

Mingfule^®^ consisted of higher single-chain content (91.2%, SD 1.3) compared with Metalyse^®^ (73.2%, SD 3.0) (Figure 3B; Supplementary Figure S2).

### 3.3 Glycosylation

#### 3.3.1 Type I/II determination: Asn184 N-glycosylation occupancy

**FIGURE 4:**
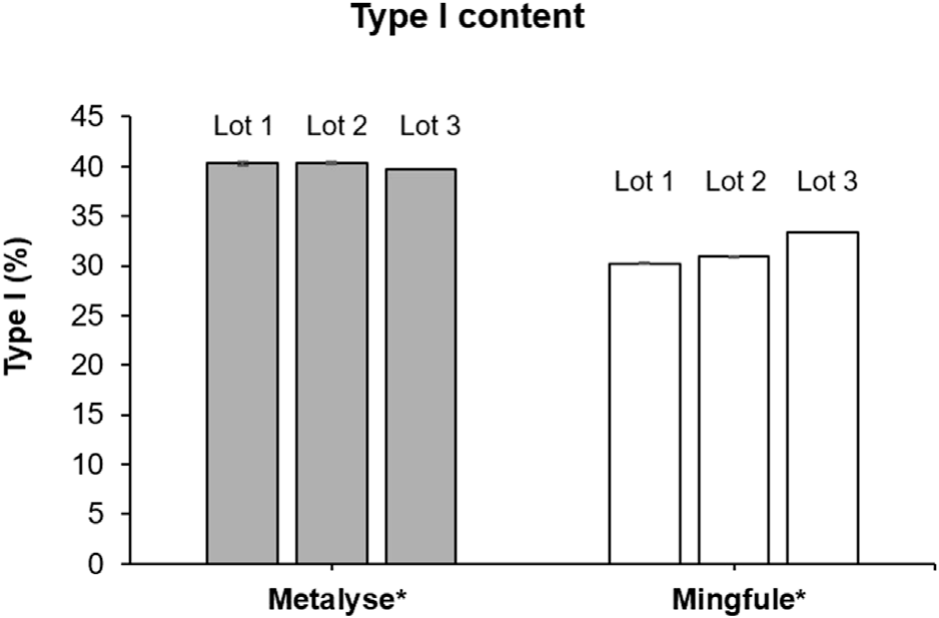
Relative abundance of type I (3 glycosylation sites occupied) forms of Metalyse^®^ and Mingfule^®^. *Each lot represents mean ± standard deviation for four replicates

The relative abundance of type I (three glycosylation sites occupied) was 40.1% (SD 0.3) and 31.5% (SD 1.4) for Metalyse^®^ and Mingfule^®^, respectively (Figure 4). The relative abundance of type II (two glycosylation sites occupied) was 59.9% (SD 0.3) and 68.5% (SD 1.4), respectively.

#### 3.3.2 N-glycosylation characterization

##### 3.3.2.1 Sialylation

Compared with Metalyse^®^, the sialylation for Mingfule^®^ was higher for the N-glycans on Asn103 (+24.5% ± 90% confidence interval 2.8%) and Asn448 (+9.0% ± 0.7%) and lower for Asn184 (−6.0% ± 1.3%) (Table 4).

**TABLE 4:** Mean offsets for post-translational modifications observed between Metalyse^®^ and Mingfule^®^. Numbers are relative to Metalyse^®^, and ±90% confidence interval.

N-glycosylation per site (%)	Asn103	Asn184	Asn448
Bi-antennary	+1.3 ± 0.7	−3.1 ± 1.0	+12.4 ± 2.6
Tri-antennary	−2.5 ± 0.4	−3.8 ± 0.5	−3.3 ± 0.9
Tetra-antennary	n.a.	−1.8 ± 0.2	−8.7 ± 1.6
Fucosylated	−35.3 ± 3.0	−9.7 ± 1.0	−1.1 ± 0.2
Mannosylated	+1.4 ± 0.1	n.a.	+0.1 ± 0.0
Galactosylated	−8.1 ± 1.7	−7.5 ± 0.6	−17.0 ± 2.1
Sialylated	+24.5 ± 2.8	−6.0 ± 1.3	+9.0 ± 0.7
O-glycosylation per site (%)			
In growth factor domain	−3.5 ± 0.5		
Additional post-translation modifications (%)			
Sum of all deamidations	+0.3 ± 0.2		
Sum of all free thiols	−75.3 ± 7.7		
Sum of all glycations	−1.6 ± 0.2		
Sum of all oxidations	−2.6 ± 1.8		
Sum of all succinimides	−0.5 ± 0.3		

Asn, asparagine.

##### 3.3.2.2 Galactosylation, fucosylation, and mannosylation

Compared with Metalyse^®^, the galactosylation for Mingfule^®^ was lower for the N-glycans on Asn103 (−8.1% ± 1.7%), Asn 184 (−7.5% ± 0.6%), and Asn448 (−17.0% ± 2.1%). Similarly, the fucosylation for Mingfule^®^ was lower for the N-glycans on Asn103 (−35.3% ± 3.0%), Asn184 (−9.7% ± 1.0%), and Asn448 (−1.1% ± 0.2%). Furthermore, for Mingfule^®^, mannosylation was marginally higher for N-glycans on Asn103 (the mean offsets were +1.4% ± 0.1%) and Asn448 (+0.1% ± 0.0%) compared with Metalyse^®^ (Table 4).

##### 3.3.2.3 Antennary branching

Compared with Metalyse^®^, the bi-antennary for Mingfule^®^ was higher for N-glycans on Asn103 (+1.3% ± 0.7%) and Asn448 (+12.4% ± 2.6%) and lower for Asn184 (−3.1% ± 1.0%). The tri-antennary for Mingfule^®^ was lower for the N-glycans at all three positions (Asn 103, -2.5% ± 0.4%; Asn 184, −3.8% ± 0.5%; Asn448, −3.3% ± 0.9%). The tetra-antennary for Mingfule^®^ was lower for N-glycans on Asn184 and Asn448 (the mean offsets were −1.8% ± 0.2% and −8.7% ± 1.6%, respectively) compared with Metalyse^®^ (Table 4).

### 3.4 Impact on clearance receptors

All three Mingfule^®^ lots showed highly dissimilar binding to the three clearance receptors compared with Metalyse^®^ (Table 2). Individual Mingfule^®^ lot similarity scores ranged from 17% to 30% for binding to LRP-1, 5% to 9% for binding to ASGR, and 8% to 10% for binding to MR.

### 3.5 Measure of heterogeneity

#### 3.5.1 Chemically induced post-translational modifications

The chemically induced post-translational modifications were relatively similar between Mingfule^®^ and Metalyse^®^, except for a pronounced difference in the abundance of free thiols (Table 4). For Mingfule^®^, the level of free thiols compared with Metalyse^®^ was −75.3% (±7.7%), indicating a substantially lower level of free thiols compared with Metalyse^®^.

#### 3.5.2 Differences in charge and size

Mingfule^®^ displayed differences in both charge and size heterogeneity compared with Metalyse^®^, displaying an average charge heterogeneity of 20.4% (SD 1.4) for region 1, 72.6% (SD 1.0) for region 2, and 6.9% (SD 0.7) for region 3. For Metalyse^®^, an average charge heterogeneity of 24.1% (SD 0.9) for region 1, 64.9% (SD 0.6) for region 2, and 11.0% (SD 0.4) for region 3 was observed. After desialylation, the charge heterogeneity of Mingfule^®^ was shown to be 15.6% (SD 0.9), 17.7% (SD 0.7), and 66.7% (SD 1.5) for regions 1, 2, and 3, respectively. Metalyse^®^ displayed a different charge distribution of 17.1% (SD 0.9), 31.1% (SD 0.7), and 51.7% (SD 1.4) for regions 1, 2, and 3, respectively, and after desialylation, CGEr revealed a comparable level of LMW species (reflected by FR1) in Mingfule^®^ compared with Metalyse^®^ (Supplementary Figure S3). As previously mentioned, HP-SEC showed that Mingfule^®^ displayed 95.7% (SD 0.3) monomer species, whereas Metalyse^®^ displayed 98.2% (SD 0.1) (Supplementary Figure S4). Furthermore, Mingfule^®^ contained a higher content of HMW (aggregate) species (3.6%, SD 0.2) compared with Metalyse^®^ (1.0%, SD 0.1).

### 3.6 Plasmin-induced activation of tenecteplase

**FIGURE 5:**
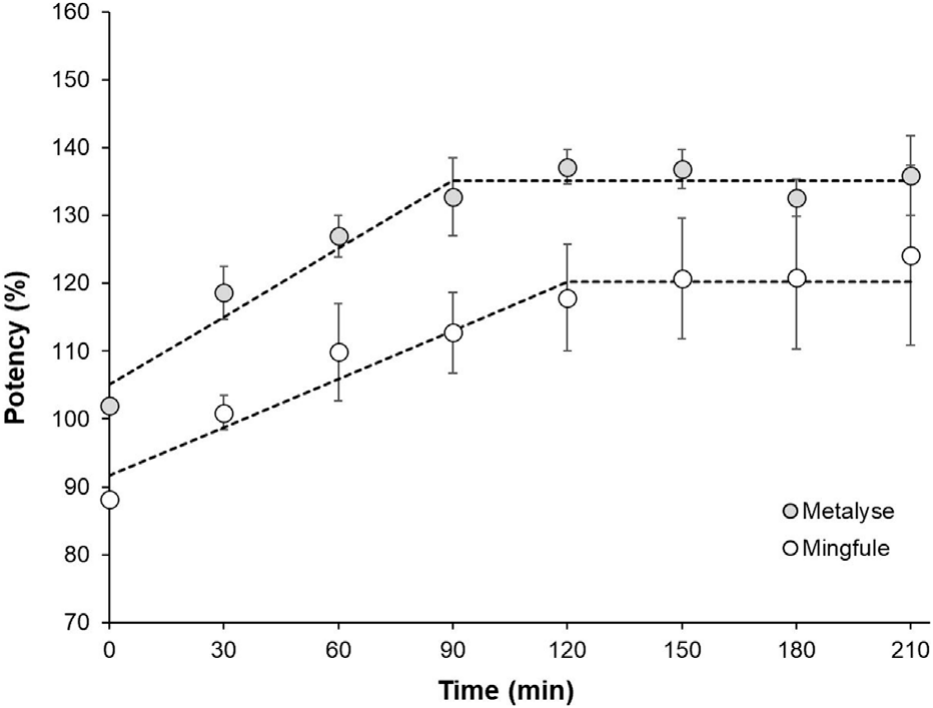
Comparison of the increase in clot lysis activity as result of plasmin-induced cleavage to the two-chain form of Metalyse^®^ and Mingfule^®^ after activation with plasmin at 37°C. Dotted line for illustration.

In contrast to Metalyse^®^, Mingfule^®^ is highly resistant to plasmin-catalyzed cleavage in its respective formulation buffer. After buffer exchange, the increase in clot lysis potency resulting from plasmin-catalyzed two-chain formation was approximately 30% slower for Mingfule^®^ in the range of linear increase. Metalyse^®^ achieved approximately 14% higher maximum clot lysis potency (Figure 5). This is in accordance with the observed difference between Metalyse^®^ and Mingfule^®^ in two-chain formation over time during incubation with plasmin.

## 4 Discussion

Here, we compared the biochemical properties and clot lysis activity of the original tenecteplase version, marketed as Metalyse^®^ and TNKase^®^, and a copy, Mingfule^®^. Metalyse^®^ is approved for the thrombolytic treatment of suspected STEMI or recent left-bundle-branch block within 6 h after the onset of acute myocardial infarction symptoms, and it is also approved for the treatment of AIS within 4.5 h of the first symptoms appearing (Boehringer Ingelheim International GmbH, 2024). Mingfule^®^ is indicated for the treatment of acute myocardial infarction, and for the fibrinolysis treatment of AIS (CSPC Pharmaceutical Group Limited, 2015; CSPC Pharmaceutical Group Limited, 2024). The current study shows that there are several significant differences between the two tenecteplase variants in terms of *in vitro* potency, purity, and biochemical makeup. The impact of these differences and how they may ultimately translate into clinical efficacy and safety can only be answered by direct comparison of the drugs in a clinical setting. The differences in products identified *in vitro* raise the question of whether data from clinical studies with one of the products can be generalized for all tenecteplase variants.

Comparative analyses revealed that the impurity profiles were less favorable for Mingfule^®^, with more aggregates and a significantly higher HCP impurity level compared with Metalyse^®^. These are key findings, as the presence of HCP impurities could potentially lead to adverse clinical effects, which may in part be attributed to HCP immunogenicity (Gutierrez et al., 2012; Jawa et al., 2016). For example, two phase III clinical trials evaluating the safety and efficacy of a recombinant biologic for the treatment of hemophilia B were suspended due to the presence of CHOP HCPs in patients treated with the drug, which clearly underscores the serious concern regulatory agencies attribute to contaminating HCPs (Ipsen, 2012).

The quantification of HCP was performed by two ELISA methods using polyclonal CHOP antibodies. It is well known that the HCP signal in ELISA shows a correlation with the amount of HCP in the sample. The ELISA signal is dependent on the specific anti-HCP antibody used in the ELISA, and therefore ELISA signals between ELISA types are variable. Although it is not possible to determine which assay provides the most accurate results without additional studies, the difference in the HCP content of Mingfule^®^ compared with Metalyse^®^ is considerable. Currently, follow-up analyses of MS-based characterization data from the HCPs detected in Metalyse^®^ and Mingfule^®^ lots are underway and may shed light on their potential impact on the quality and performance of these drugs.

In terms of specific impurities, clusterin, a glycoprotein associated with clearance of cellular debris and apoptosis, was detected in all Mingfule^®^ lots but was not detected in those of Metalyse^®^. Clusterin has been shown to non-specifically interact with monoclonal antibodies (mAbs) and has been detected post-protein A purification of multiple mAb products. It is considered a high-risk HCP due to its potential to cause unwanted immune responses in patients and, in turn, may impact drug quality (Zhang et al., 2014; Jawa et al., 2016).

Analyses of post-translational modifications and sialylation patterns also differed between Mingfule^®^ and Metalyse^®^, with potential implications for the pharmacokinetic/pharmacodynamic profile. There was a difference displayed in antennary branching, with the overall bi-antennary higher for Mingfule^®^, whereas the overall tri- and tetra-antennary were higher for Metalyse^®^; differences in antennary have the potential to impact clearance of glycopeptides (Baenziger and Fiete, 1980). There were also differences in the relative abundance of type I (three glycosylation sites occupied) and type II (two glycosylation sites occupied) molecules based on Asn184 N-glycosylation occupancy, with Metalyse^®^ displaying a higher type I content. Increased type I has been shown to result in reduced clearance/increased half-life of tenecteplase, and may thereby facilitate its administration as a single bolus instead of a bolus accompanied by continuous infusion (Cole et al., 1993; Wang et al., 2024). The free thiol Cys83 may influence the binding affinity of fibroblast growth factor (FGF) proteins to LRP-1 by modulating the conformation and stability beta-trefoil domain of FGF proteins (Vandooren and Itoh, 2021).

Dissimilar fibrin binding affinity of Mingfule^®^ and Metalyse^®^ was observed, as well as a high degree of dissimilarity in the binding to clearance receptors LRP-1, ASGR, and MR. Differences in fibrin binding may be relevant to pharmacodynamics as well as potency, whereas affinity to the clearance receptors may potentially impact on half-life. Differences in galactosylation and sialylation can impact ASGR binding and clearance rates. ASGR and the MR (C-type 1) are well known for their selective recognition, and clearance of circulating glycoproteins and asialylated glycoproteins is selective (Fukuda et al., 1989; Stockert, 1995). Sialylation of the glycoprotein, however, prevents recognition by ASGR and reduces clearance. In this context, differences in galactosylation and sialylation were noted between Mingfule^®^ and Metalyse^®^, which are expected to translate into the observed dissimilarity in ASGR binding. In addition, mannosylation, which can impact MR binding, was higher for Mingfule^®^, but the absolute values remained close to negligible.

Therefore, glycosylation is a critical attribute that can modulate the efficacy of a commercial therapeutic glycoprotein by balancing activity and clearance. Assuring a consistent and specific N-glycan, consisting of a spectrum of product glycans, can be crucial to achieve the desired therapeutic efficacy.

For a successful thrombolytic therapy, a fine balance between activity and half-life is essential. This fine balance, resulting in a favorable safety profile, has been demonstrated for Metalyse^®^ since 2001, but the observed differences mean that this cannot be assumed for any tenecteplase copy without the generation of additional clinical evidence in relevant patient populations. That said, clinical studies with Mingfule^®^ in China have provided the basis for regulatory approval in China, with the first indication in 2015 (European Medicines Agency, 2024; CSPC Pharmaceutical Group Limited, 2024).

Fundamentally, *in vitro* clot lysis potency was 13.5% lower for Mingfule^®^ (84.8%) than Metalyse^®^ (98.3%), potentially driven by the proportion of single-chain content of Mingfule^®^ (91.2%) compared with Metalyse^®^ (73%), as well as differences in sialic acid content and purity. Different neurologic effects of single-chain (sc-) and two-chain (tc-) t-PA have previously been discussed and are likely to have similar implications for tenecteplase. Using clinical and preclinical data, Anfray et al. (2021) demonstrated that the impact of rt-PA in stroke patients may be dependent on which form of rt-PA is administered (sc-rt-PA *versus* tc-rt-PA) (Anfray et al., 2021). It should be noted that conversion of sc-t-PA to tc-t-PA does not occur at physiologically relevant rates in plasma (Rijken et al., 1982). Thus, different levels of single-chain are expected to have different effects outside the blood clot. In this context, interaction with different receptors might be of relevance. *In vitro* analysis showed that the intrinsic activity of sc-t-PA selectively modulates N-methyl-D-aspartate receptor (NMDAR) signaling compared with tc-t-PA (Bertrand et al., 2015). tc-t-PA activates the MET receptor, leading to the formation of proximal complexes between MET and NMDARs, and down-regulation of glutamate ionotropic receptor NMDA-type subunit 2-NMDAR-driven signaling, whereas sc-t-PA promotes the disruption of these complexes, potentially improving neuronal survival (Bertrand et al., 2015; Hedou et al., 2021). The physiologic effect of differences in sc-t-PA and tc-t-PA ratios is not yet fully understood. Especially for tenecteplase, available literature is limited.

High single-chain content may impact potency *in vivo*, as the full activation (or conversion to two-chain) of tenecteplase may only be achieved in a timeframe when lysis of most blood clots would have already happened. The conversion speed of sc-t-PA to tc-t-PA in the presence of purified fibrin clots is similar in scale to the duration of recanalization in AIS treatment with tenecteplase (Tsivgoulis et al., 2020). Compared with Metalyse^®^, the *in vitro* increase in potency by plasmin-catalyzed two-chain formation of Mingfule^®^ happened at an approximately 30% slower rate. This may also lead to reduced clot lysis *in vivo*. The root cause for the slower increase in activity is not well understood but was observed in the past for other tenecteplase products with differing quality profiles (Blandizzi et al., 2017), and thus might be linked to post-translational modifications. Additionally, it is known that the content of sialic acid residues affects the biological activity of tenecteplase (Chaschinova et al., 2020), and it is therefore feasible that the difference in sialylation of Mingfule^®^ compared with Metalyse^®^ may contribute to its lower clot lysis potency.

Overall, the evaluation of similarity between a non-innovator biological product and its originator drug is a multifaceted process that requires pharmaceutical, non-clinical, and clinical comparative studies. The pharmaceutical comparison results should guide the design of subsequent studies, aiming to resolve any uncertainties and support the overall similarity evaluation. In this context, the tenecteplase copy, Mingfule^®^, has been shown to have several meaningful differences from the originator, Metalyse^®^. Therefore, with a comprehensive understanding of the clinical implications, healthcare professionals can make informed decisions regarding the generalizability of clinical results generated with different tenecteplase products.

## Data Availability

The datasets used and/or analyzed during the current study are available from the study sponsor, Boehringer Ingelheim, on reasonable request. To ensure independent interpretation of clinical study results and enable authors to fulfil their role and obligations under the ICMJE criteria, Boehringer Ingelheim grants all external authors access to clinical study data pertinent to the development of the publication. In adherence with the Boehringer Ingelheim Policy on Transparency and Publication of Clinical Study Data, scientific and medical researchers can request access to clinical study data when it becomes available on Vivli - Center for Global Clinical Research Data, and earliest after publication of the primary manuscript in a peer-reviewed journal, regulatory activities are complete, and other criteria are met. Please visit for further information: https://www.mystudywindow.com/msw/datasharing.
