# Supplementary material for: Tenecteplase: biochemical and clot lysis activity comparisons
Source: Front Pharmacol. 2024 Dec 20;15:1498116. doi: 10.3389/fphar.2024.1498116 (PMC11695638; doi:10.3389/fphar.2024.1498116)
Supplement: Supplementary file 1 [file Image4.pdf]

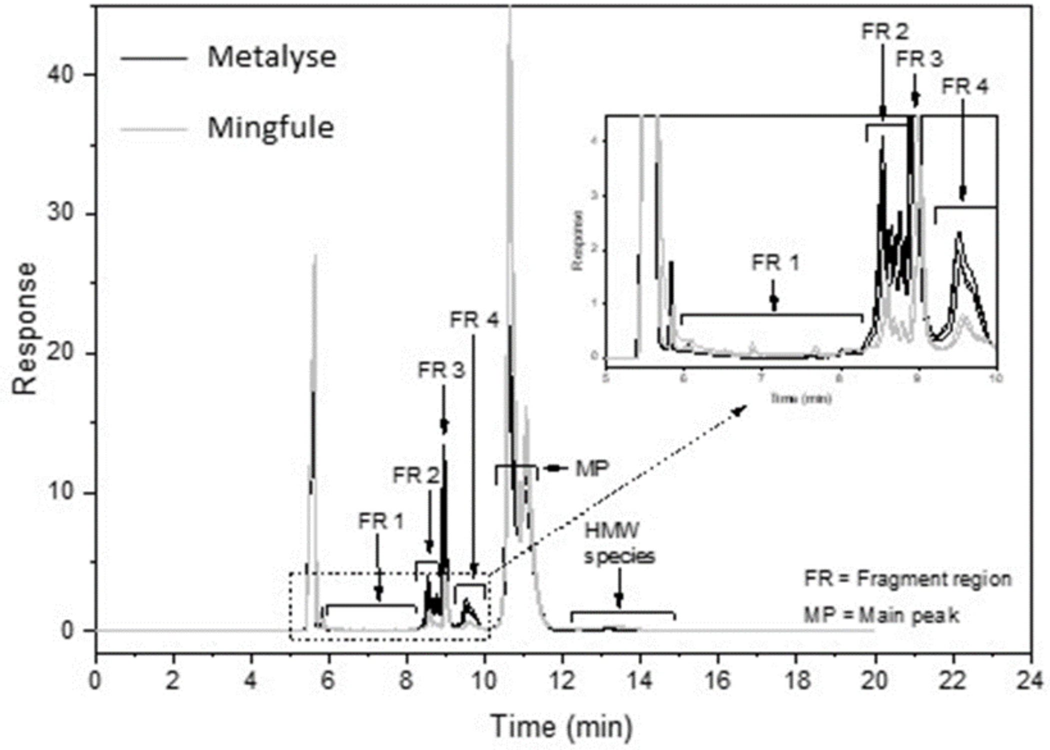

Figure S4. CGE reduced electropherograms (side-by-side testing) including enlarged scale.

CGE, capillary gel electrophoresis; HMW, high-molecular-weight.
