# Supplementary figures and images for: Tenecteplase: biochemical and clot lysis activity comparisons
Source: Front Pharmacol. 2024 Dec 20;15:1498116. doi: 10.3389/fphar.2024.1498116 (PMC11695638; doi:10.3389/fphar.2024.1498116)

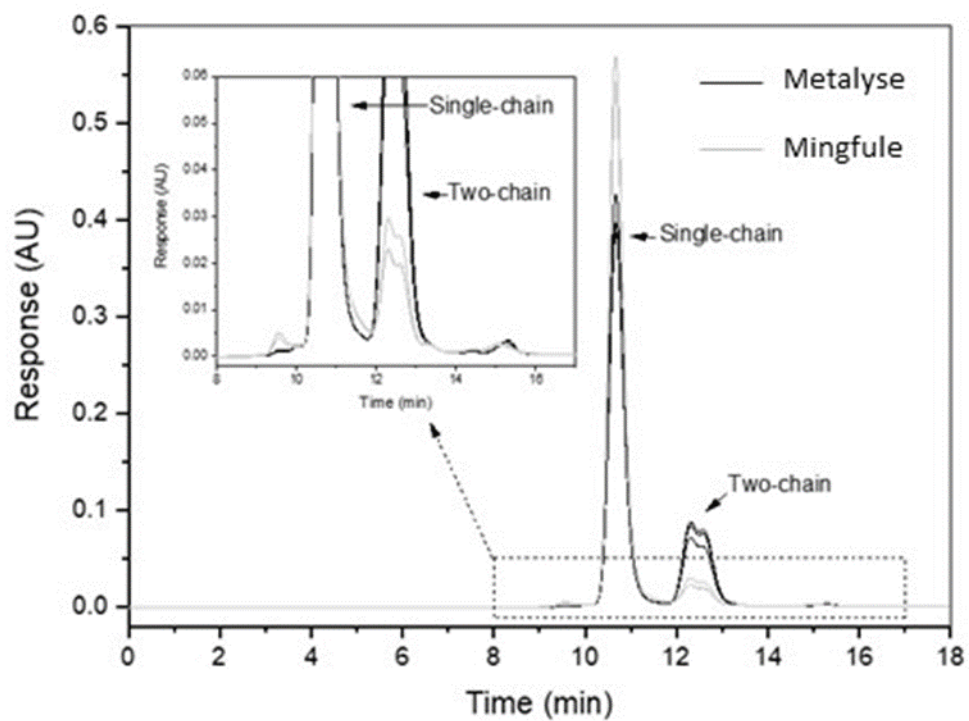

**Figure S2.** Single-chain chromatograms (side-by-side testing) including enlarged scale.

Supplement: Supplementary file 2 [file Image2.pdf]
