# Supplementary material for: Tenecteplase: biochemical and clot lysis activity comparisons
Source: Front Pharmacol. 2024 Dec 20;15:1498116. doi: 10.3389/fphar.2024.1498116 (PMC11695638; doi:10.3389/fphar.2024.1498116)
Supplement: Supplementary file 3 [file Image3.pdf]

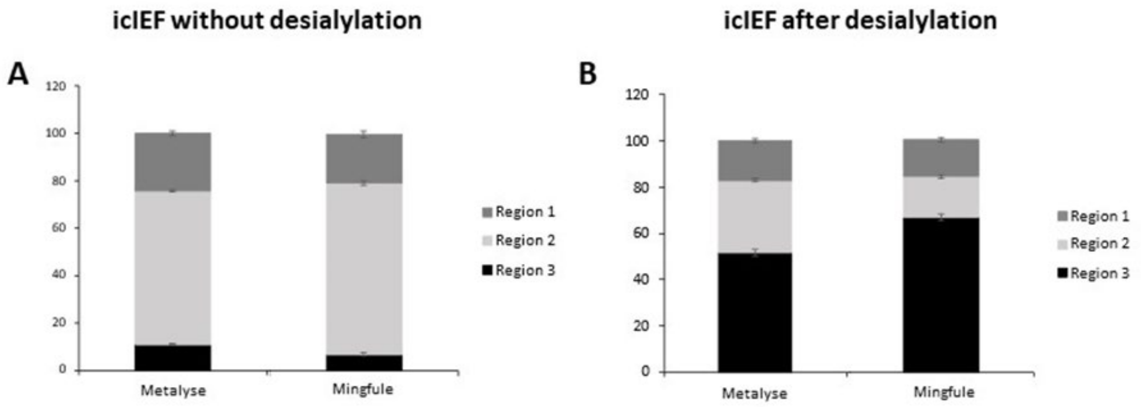

Figure S3. Differences in charge heterogeneity. **(A)** icIEF without desialylation, **(B)** icIEF with desialylation.

icIEF, imaged capillary isoelectric focusing.
