# Supplementary material for: Tenecteplase: biochemical and clot lysis activity comparisons
Source: Front Pharmacol. 2024 Dec 20;15:1498116. doi: 10.3389/fphar.2024.1498116 (PMC11695638; doi:10.3389/fphar.2024.1498116)
Supplement: Supplementary file 4 [file Image1.PDF]

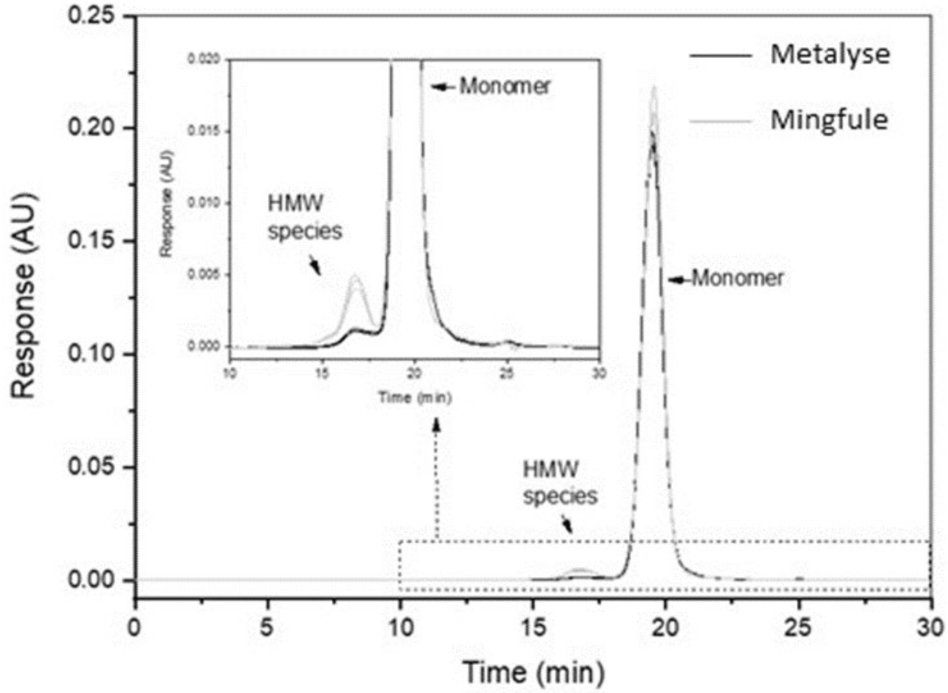

Figure S1. HP-SEC chromatograms (side-by-side testing) including enlarged scale.

HMW, high-molecular-weight; HP-SEC, high-performance size exclusion chromatography.
